# The Nutritional Supply of Iodine and Selenium Affects Thyroid Hormone Axis Related Endpoints in Mice

**DOI:** 10.3390/nu13113773

**Published:** 2021-10-25

**Authors:** Kristina Lossow, Kostja Renko, Maria Schwarz, Lutz Schomburg, Tanja Schwerdtle, Anna Patricia Kipp

**Affiliations:** 1 Department of Nutritional Physiology, Institute of Nutritional Sciences, Friedrich Schiller University Jena, 07743 Jena, Germany; kristina.lossow@uni-jena.de (K.L.); schwarz.maria@uni-jena.de (M.S.); 2 TraceAge-DFG Research Unit on Interactions of Essential Trace Elements in Healthy and Diseased Elderly, 07743 Jena, Germany; lutz.schomburg@charite.de (L.S.); tanja.schwerdtle@bfr.bund.de (T.S.); 3 German Institute of Human Nutrition, 14558 Nuthetal, Germany; 4 German Federal Institute for Risk Assessment (BfR), 12277 Berlin, Germany; Kostja.Renko@bfr.bund.de; 5 Institute for Experimental Endocrinology, Charité-University Medical School Berlin, 13353 Berlin, Germany; 6 Department of Food Chemistry, Institute of Nutritional Science, University of Potsdam, 14558 Nuthetal, Germany

**Keywords:** selenium, iodine, thyroid, kidney, liver, mouse

## Abstract

Selenium and iodine are the two central trace elements for the homeostasis of thyroid hormones but additional trace elements such as iron, zinc, and copper are also involved. To compare the primary effects of inadequate intake of selenium and iodine on the thyroid gland, as well as the target organs of thyroid hormones such as liver and kidney, mice were subjected to an eight-week dietary intervention with low versus adequate selenium and iodine supply. Analysis of trace element levels in serum, liver, and kidney demonstrated a successful intervention. Markers of the selenium status were unaffected by the iodine supply. The thyroid gland was able to maintain serum thyroxine levels even under selenium-deficient conditions, despite reduced selenoprotein expression in liver and kidney, including deiodinase type 1. Thyroid hormone target genes responded to the altered selenium and iodine supply, whereas the iron, zinc, and copper homeostasis remained unaffected. There was a notable interaction between thyroid hormones and copper, which requires further clarification. Overall, the effects of an altered selenium and iodine supply were pronounced in thyroid hormone target tissues, but not in the thyroid gland.

## 1. Introduction

Selenium and iodine are two important representatives of the group of essential trace elements with well-known impact on health status of the mammalian organism. While selenium exerts its effect primarily via selenoproteins [1], iodine acts exclusively as part of the iodine-containing thyroid hormones (TH) triiodothyronine (T3) and thyroxine (T4) as well as its metabolites [2]. TH regulate a variety of processes, including growth, development, and metabolic rate. Within the mammalian organism, TH are exclusively synthesized de-novo by the thyroid gland, secreting the prohormone T4 as main product and, to a lesser extent, the active hormone T3. Regarding the biological activity T4 ranges 10-fold lower than T3, when compared in regard to TH receptor binding affinity [3].

Worldwide, there are still areas where the intake of either or both trace elements is low [4,5]. For iodine, extensive efforts have been undertaken in numerous countries to improve the daily intake by mandatory iodine fortification of foods for example iodination of table salt [4,6]. Factors that play a decisive role in the supply of these nutrients include deterioration of soil conditions [7], which affects the content of food, but also the increasing prevalence of dietary habits that may increase the risk of inadequate iodine and selenium intake, particularly in Europe. Vegetarians and in particular vegans are at risk to develop a deficiency of iodine and selenium or even both [8,9,10,11]. In addition to diet, pathophysiological conditions such as inflammatory bowel disease or renal insufficiency may increase compromised status due to disturbed absorption, metabolism, and excretion of trace elements, respectively.

The thyroid gland is able to accumulate iodine and selenium actively. It has the highest tissue concentrations of both trace elements among all organs of the human body [12]. Decades ago, it was observed that selenium is needed in addition to iodine for optimal thyroid function [13]. Selenoproteins are involved in two important processes controlling TH biosynthesis and activity: (i) deiodinases (DIO) are essential for the local activation and inactivation of TH; (ii) excessive hydrogen peroxide, actively generated to enable the coupling of iodide to thyroglobulin in order to produce TH, has to be safely degraded. This reaction is to a certain extend catalysed by glutathione peroxidases (GPX) and thioredoxin reductases (TXNRD) as well as by selenoprotein P (SELENOP) [14]. Animal experiments have indicated that a lack of selenoproteins in thyroid epithelial cells results in more lipid peroxidation and nitrosative stress [15]. Such selenium-dependent effects are supposed to be involved in the etiology of myxedematous cretinism, a severe form of endemic cretinism found in central Africa [16,17]. Vice versa, some pathological changes by selenium deficiency can be aggravated by iodine deficiency, thereby affecting growth and bone structure [18,19]. In addition, DIO are needed in TH target tissues for example liver or kidney for local regulation of intracellular TH levels. By releasing iodine from specific positions of the TH core structure, T4 can be converted to the active T3 but also to the inactive metabolite reverse T3 (rT3). This system of specific activation and inactivation on the cellular level provides an important mechanism for pre-receptor control of TH activity. In contrast to the thyroid gland, most “peripheral” organs such as liver and kidney are more sensitive towards changes in the selenium supply due to organ hierarchy for selenium, suggesting that DIO activity might be affected by suboptimal supply.

In addition to iodine and selenium, also other trace elements such as iron, zinc, and copper are needed for proper thyroid function. Iron deficiency, the most common global trace element deficiency, adversely affects heme-containing thyroperoxidase (TPO), which catalyses initial steps of TH synthesis [20,21,22]. Zinc serves as a co-factor for numerous enzymes and is crucial in transcriptional regulation, being an essential part of for example zinc finger proteins involved in DNA-binding and promoter regulation. Direct effects of altered Zn supply on the TH axis have been reported for example for thyrotropin releasing hormone (TRH) and thyroid stimulation hormone (TSH) concentrations as well as deiodinase activity [23,24,25,26]. Furthermore, the nuclear receptors for T3 (thyroid hormone receptor, THR) contain two Zn finger motifs that are involved in dimerization and DNA binding [27,28,29]. Also, copper is associated with TH as the expression of some copper-dependent genes is regulated by THR [30].

Accordingly, the interactions of trace elements with respect to thyroid function, TH biosynthesis, and/or TH action on target organs are complex. Vice versa, iodine status might affect selenium metabolism directly or indirectly due to functional interrelation. Here, we aimed to evaluate feeding-induced effects of low and adequate amounts of selenium and iodine to elucidate interactions of both trace elements considering the organ hierarchy for selenium in the mouse. Hereby, we want to elucidate how a low selenium supply affects the thyroid gland itself but also target tissues of TH, both under euthyroid or mild hypothyroid conditions induced by the limited iodine supply. In addition, interactions of the selenium and iodine supply with the homeostasis of other crucial trace elements involved in TH synthesis, namely iron, copper, and zinc, are analysed.

## 2. Materials and Methods

### 2.1. Animal Husbandry

Animal experiment was approved by and conducted following national guidelines of the Ministry of Environment, Health and Consumer Protection of the federal state of Brandenburg, Germany (2347-44-2017) and institutional guidelines of the German Institute of Human Nutrition Potsdam-Rehbruecke, Germany.

C57BL/6Jrj mice were housed in small groups at constant regulations with 12:12 h light:dark cycle, 22 °C room temperature, and 55% humidity. With the age of three weeks (after weaning) mice received a torula-based diet (modified C1045, Altromin, Lage, Germany) low in selenium, iodine, iron, and copper (0.02, 0.03, 17.2, and 3.65 mg/kg, respectively).

Mice were divided into four groups of 7 animals each, varying with regard to their selenium and iodine supply. Accordingly, mice were either supplied adequately with both elements (+Se/+I), adequately with selenium but insufficient with iodine (+Se/−I), deficient in selenium but adequately with iodine (−Se/+I) or deficient in both elements (−Se/−I). The distinction in adequate and inadequate supply is based on the feeding recommendation for mice [31], which were undercut when the animals were exclusively supplied with selenium and iodine via the diet. Therefore, selenium (0.13 mg selenium/kg; Na_2_SeO_3_, Thermo Fisher Scientific, Waltham, USA) and iodine (0.15 mg iodine/kg; KI, Sigma-Aldrich/Merck, Darmstadt, Germany) were added to the drinking water to achieve adequate supply. For adequate iron supply, drinking water of all mice was further fortified with 17.8 mg iron/kg (FeCl_2_, Merck, Germany). More detailed information can be found in Table 1. The dietary intervention lasted for 8 weeks with food and water offered *ad libitum*. Finally, mice were anesthetized with isoflurane (Cp-pharma, Burgdorf, Germany), followed by blood withdrawal by heart puncture. Serum was obtained after full coagulation at room temperature (RT) and centrifugation for 10 min (3000× *g*, 4 °C). Organs were surgically dissected, snap-frozen in liquid nitrogen, and stored at −80 °C until further use.

### 2.2. Analysis of Thyroid-Axis Related Enzyme Activities

Frozen specimens were powdered, mixed with homogenization buffer (250 mM D-Sucrose, 20 mM Hepes, 1 mM EDTA, pH 7.4) and underwent subsequent sonication (2×10 pulses, UP50H, Hielscher, Teltow, Germany). Homogenates were adjusted to a uniform concertation (liver and kidney) and a total of 40 µg protein was added to each reaction.

For thyroid lobes, the tissues were minced in 10 mM Tris-HCl (pH 7) via micropestle. Subsequently, tissue homogenate was mixed with an equal amount of homogenization buffer and further diluted to achieve similar concentrations for each sample (0.5 µg/µL). A total of 20 µg protein was added to each reaction.

Relative Dio1 activity was determined essentially as described before with slight modifications [32]. Samples (two technical replicates) were mixed with a respective master mix to achieve assay conditions (final concentrations: 100 mM KPO_4_ pH 6.8, 1 mM EDTA, 40 mM DTT, 10 µM rT3) and incubated for two hours at 37 °C under constant shaking (500 rpm). Subsequently, 75 µL were transferred to a microtiter column pack (600 µL DOWEX W50× 2 per column, NUNC, Thermo Fisher Scientific) and released iodine in the form of iodide was eluted after addition of 100 µL acetic acid (10%) via centrifugation.

The eluted iodine was quantified by using the Sandell-Kolthoff-reaction [32,33]. 50 µL of further diluted eluate was transferred to a microtiter plate (TPP, Trasadingen, Switzerland). Reaction was started by adding Ce-solution (25 mM (NH_4_)_4_Ce(SO_4_)_4_ and 0.5 M H_2_SO_4_) and As-solution (25 mM NaAsO_2_, 0.5 M H_2_SO_4_, 0.2 M NaCl) and destaining was recorded at the beginning and after 20 min at 416 nm in a respective plate photometer at room temperature. After subtraction of background (average from four samples with 1 mM of propylthiouracil as Dio1-inhibitor), a separately measured iodine standard curve (KI standard) was used for interpolation and approximation of iodine release.

For the analysis of iodotyrosine deiodinase (Dehal1) activity, homogenates of liver, kidney, and thyroid tissue were prepared as described above. Relative Dehal1 activity was determined essentially as described before [34] with slight modifications. Samples (two technical replicates) were mixed with a respective master mix to achieve assay conditions (final concentrations: 100 mM KPO_4_ pH 7, 200 mM KCl, 10 mM β-mecaptoethanol, 0.8 mM NADPH, 30 µM FAD, 10 µM MIT) and incubated for four hours at 37 °C under constant shaking (500 rpm). Subsequently, 75 µL were transferred on a microtiter column pack (600 µL DOWEX W50×2 per column, NUNC) and released iodine in the form of iodide was eluted after addition of 100 µL acetic acid (10%) via centrifugation.

The eluted iodine was quantified by using the Sandell-Kolthoff-reaction as described above. In this case, an average background signal was acquired by four samples with 1 mM of 3,5-dibromotyrosine as Dehal1 inhibitor.

Thyroidal peroxidase activity was measured by utilizing Amplex-UltraRed fluorescent dye (Invitrogen, Thermo Fisher Scientific) as substrate, essentially adapting the principle described for respective *in-vitro* assays in the context of inhibitor screenings [35]. Equal amounts of thyroid tissue homogenate (total of 5 µg protein in 10 mM Tris-HCl) were transferred to a black microtiter plate (Brand, Wertheim, Germany). For each sample, two technical replicates were used for relative activity determination while one replicate was inhibited by addition of thiamazole (MMI) as TPO inhibitor (1 mM). Reaction was started by adding a master mix to reach assay conditions (100 mM KPO_4_ pH 7, 50 µM Amplex-UltraRed, 0.0035% H_2_O_2_) and subsequent incubation for 10 min at room temperature. After shaking, fluorescence was recorded (EX:535 nm EM:590 nm) as readout for peroxidase activity within the samples in a respective plate reader (GENIOS, Tecan, Männedorf, Switzerland). The signal (RFU) from MMI-inhibited samples was subtracted from the respective uninhibited samples to calculate MMI-sensitive peroxidase activity.

### 2.3. TE Analysis in Serum, Tissue, and Diet

For TE analysis pulverized frozen tissue and feed samples were subjected to acidic microwave digestion as reported before [36]. After dilution resulting in a final concentration of 2.93% HNO_3_. Serum samples were diluted 1:10 prior analysis by ICP-MS/MS (8800 ICP-QQQ-MS, Agilent Technologies, Waldbronn, Germany). Details were described elsewhere [37,38].

For the analysis of the relative thyroidal iodine content, 10 µL of tissue homogenate (protein concentration of 0.25 µg/µL) was mixed with 50 µL of 0.6 M ammonium persulfate for oxidative digestion and release of bound iodine. Samples were incubated for 1 h at 95 °C in a Thermocycler (Eppendorf, Hamburg, Germany). Subsequently, 50 µL of each sample was transferred in a respective multiwell plate (TPP). Relative iodine content was determined by the Sandell-Kolthoff-reaction (see above). Here, changes in absorption (delta OD) underwent no further calculation but were used to directly demonstrate the qualitative changes of iodine content within the thyroid tissue by the respective diets.

### 2.4. Selenop Analysis in Serum

Selenop concentration in serum was determined by affinity high performance liquid chromatography coupled to inductively coupled plasma mass spectrometry (affinity-HPCL-ICP-MS/MS) as reported before [39].

### 2.5. Determination of Serum Parameters by ELISA or Multiplex Assay

Total T3 (tT3) and T4 (tT4) levels as well as transferrin concentrations in serum were analyzed applying an enzyme-linked immunosorbent assay kit (tT3 and tT4: Abnova Corporation, Taipei City, Taiwan; transferrin: Abcam, Cambridge, UK). To this end, serum samples were either applied undiluted (tT3), after a 1:2 (tT4) or 1:200,000 dilution (transferrin) to the microplate, following manufacturer’s instructions. The average absorbance was measured at 450 nm using a microplate reader (Synergy H1, BioTek, Bad Friedrichshall, Germany). Calculation of concentrations was performed based on standard curves.

TSH levels in serum were measured by using the Mouse Pituitary Magnetic Bead Panel-Endocrine Multiplex Assay (MPTMAG-49K, Millipore, Darmstadt, Germany) and a Luminex 200 device, based on the Luminex xMAP technology. Assay was performed in accordance with the manual. Average CV [%] of all included samples was <10%.

### 2.6. Analysis of Enzyme Activities

Frozen tissue samples were homogenized in Tris buffer (100 mM Tris (Carl Roth), 300 mM KCl (Applichem, Darmstadt, Germany), 0.1% (*v*/*v*) Triton X-100, (Serva, Heidelberg, Germany) with protease inhibitor (1 µL/mL (*v*/*v*); Merck/Millipore, Burlington, VT, USA, pH 7.6) followed by centrifugation for 10 min at 14,000 g and 4 °C. Received tissue samples free of cellular debris were directed to measurement of GPX [40] and TXNRD [41] based on NADPH-consuming glutathione reductase-coupled assay and NADPH-dependent reduction of 5,5′-dithiobis (2-nitrobenzoic acid) (DTNB) as previously described. All measurements were performed in triplicates using a microplate reader (Synergy H1). Enzymatic activities were normalized to protein content based on Bradford analysis (Bio-Rad Laboratories, Munich, Germany).

### 2.7. PCR Analysis of the Liver, Kidney, and Pituitary Gland

Total RNA was isolated according to Trizol Reagent manufacturer’s instructions (Invitrogen). 9.0, 7.0, and 0.3 µg liver, kidney, and pituitary RNA, respectively, were subjected to PerfeCTa DNase I (Quanta BioSciences, Beverly, USA) digestion followed by reverse transcription in a final volume of 20 µL (qScript cDNA synthesis, Quanta BioSciences). For complementary DNA (cDNA) amplification, 1x PerfeCTa SYBR Green Supermix (Quanta BioSciences), cDNA, and 250 nM oligonucleotides (sequences are listed in Table S1) were mixed in a total volume of 10 µL following the thermal cycling program consisting of 3 min at 95 °C, followed by 41 cycles of 15 s at 95 °C, 20 s at 50 to 60 °C (for details see Supplementary Table S1), and 30 s at 72 °C. Quantitative real-time polymerase chain reaction (qRT-PCR) was performed using Mx3005P real-time PCR system (Stratagene, Agilent Technologies). Copy numbers were calculated based on standard curves. Expression levels were normalized to a composition factor based on the housekeeper genes hypoxanthine phosphoribosyltransferase 1 (Hprt), ribosomal protein L13a (Rpl13a), 18S ribosomale RNA, beta-actin, TATA-binding protein (TBP), and glyceraldehyde 3-phosphate dehydrogenase (Gapdh) for liver and kidney samples or Hprt and Rpl13a for the pituitary glands.

### 2.8. Statistical Analysis

Statistical analysis was performed with GraphPad Prism 9 (GraphPad Software, La Jolla, CA, USA). After removal of outliers based on Grubb’s Test, Two-Way analysis of variance (ANOVA) followed by Bonferroni’s post-test was conducted. The calculation of correlation coefficient was performed according to Spearman (referred to as r_s_). Statistical significances are shown as */^#^ *p* < 0.05, **/^##^ *p* < 0.01, ***/^###^ *p* < 0.001.

## 3. Results

### 3.1. Biomarkers of Iodine Status in Relation to Iodine and Selenium Supply

To study putative interactions of selenium and iodine, C57BL/6Jrj mice were fed a torula yeast-based diet with either low or adequate selenium and iodine supply for a period of 8 weeks. Although the intervention already started in the third week of life and the supply with trace elements is supposed to have an effect on growth and development, no adverse effects could be observed with regard to body weight (Figure S1A) and relative organ weights (Figure S1B–G). Only for the relative kidney weights a trend (*p* = 0.094) for an iodine-dependent reduction was found in mice with an inadequate iodine supply (Figure S1C).

**Figure 1:**
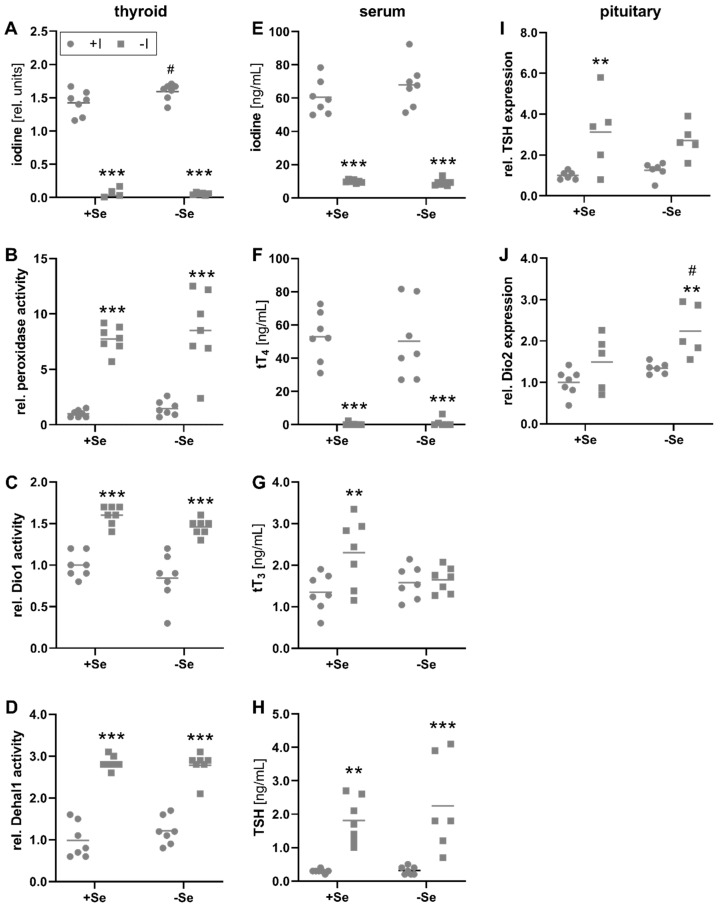
Effects of the iodine and selenium supply on biomarkers of the iodine status. Concentrations, activities, and expression levels of biomarkers reflecting the iodine status of male C57BL/6Jrj mice (*n* = 5–7) were determined after receiving either low or adequate concentrations of selenium (Se) and iodine (I) over 8 weeks. Therefore, thyroidal iodine content (**A**) and activity of enzymes involved in the formation and/or degradation of TH, such as MMI-sensitive peroxidase activity (**B**), Dio1 (**C**), and Dehal1 (**D**) were quantified by Amplex-UltraRed oxidation or Sandell-Kolthoff-based assays, respectively. Further, total serum iodine (**E**), tT4 (**F**), tT3 (**G**), and TSH (**H**) concentrations were determined by using ICP-MS/MS, ELISA, and xMAP technology, respectively. Negative feedback mechanisms were addressed by measurement of TSH (**I**) and Dio2 (**J**) expression within pituitary glands by qRT-PCR. Expression levels were normalized to a composition factor based on the housekeeping genes Hprt and Rpl13a. For enzyme activities and expression analysis, results are shown as fold change relative to +Se/+I-supplied animals. Statistical testing based on Two-way ANOVA with Bonferroni’s post-test with ** *p* < 0.01; *** *p* < 0.001 versus + iodine and ^#^ *p* < 0.05 versus + selenium.

The iodine content of the thyroid gland in iodine-deficient animals was almost undetectable under the given methodological limitations, in contrast to the adequately supplied group, demonstrating the successful application of the dietary protocol (Figure 1A). Based on these technical limitations, it is not possible to draw any conclusions about selenium dependencies under iodine deficiency. However, the relative thyroidal iodine content of iodine-adequate animals appeared to be slightly increased by selenium. MMI-sensitive peroxidase activity measured by Amplex-UltraRed oxidation, was inversely regulated, with approximately 7-times higher activities in iodine-deficient animals (Figure 1B). While this readout is highly experimental due to the unspecific character of the used substrate, the measured activity could represent TPO catalyzing the iodination and coupling of tyrosyl residues to thyreoglobulins to form TH and, as indicated by the data, might represent the activation status of the thyroid tissue, which is stimulated in situations of iodine deficiency by elevated TSH. Comparably, the thyroidal activities of Dio1 and Dehal1, responsible for the deiodination of TH and the recycling of iodine, respectively, revealed a comparable dependency on the iodine intake (Figure 1C, D). However, there was no effect of selenium on either of these three enzyme activities in the thyroid tissue (Figure 1B–D).

The iodine deficiency which was detected on the thyroid level was also reflected by the total iodine concentrations of the serum. Here, low iodine intake resulted in 7-times lower values than an adequate iodine supply (Figure 1E). In line with this, the hormone T4 showed a clear dependence on the iodine intake and is supposed to be the main contributor to serum iodine concentrations (Figure 1F). The varying selenium supply had no effect on serum iodine or T4 concentrations. In contrast, the biologically more active T3 was increased in the serum of +Se/−I-supplied in comparison to +Se/+I-supplied mice but unaffected by the iodine status in selenium-deficient mice (Figure 1G). Thus, serum T3 levels tended to be higher in +Se/−I-supplied animals compared to −Se/−I-supplied mice (*p* = 0.06). Differences in circulating TH levels also affect the negative feedback mechanisms of the hypothalamus-pituitary gland axis. Serum TSH concentrations were significantly increased in animals receiving an insufficient iodine supply (Figure 1H). Accordingly, animals with low iodine supply showed higher TSH expression in the pituitary (Figure 1I). The mRNA expression of Dio2 in the pituitary was increased in the −Se/−I-supplied group revealing a cumulative iodine and selenium effect in comparison to the respective adequately supplied group (+Se/+I, +Se/−I; Figure 1J).

### 3.2. The Systemic Selenium Status Is Unaffected by Iodine Deficiency

**Figure 2:**
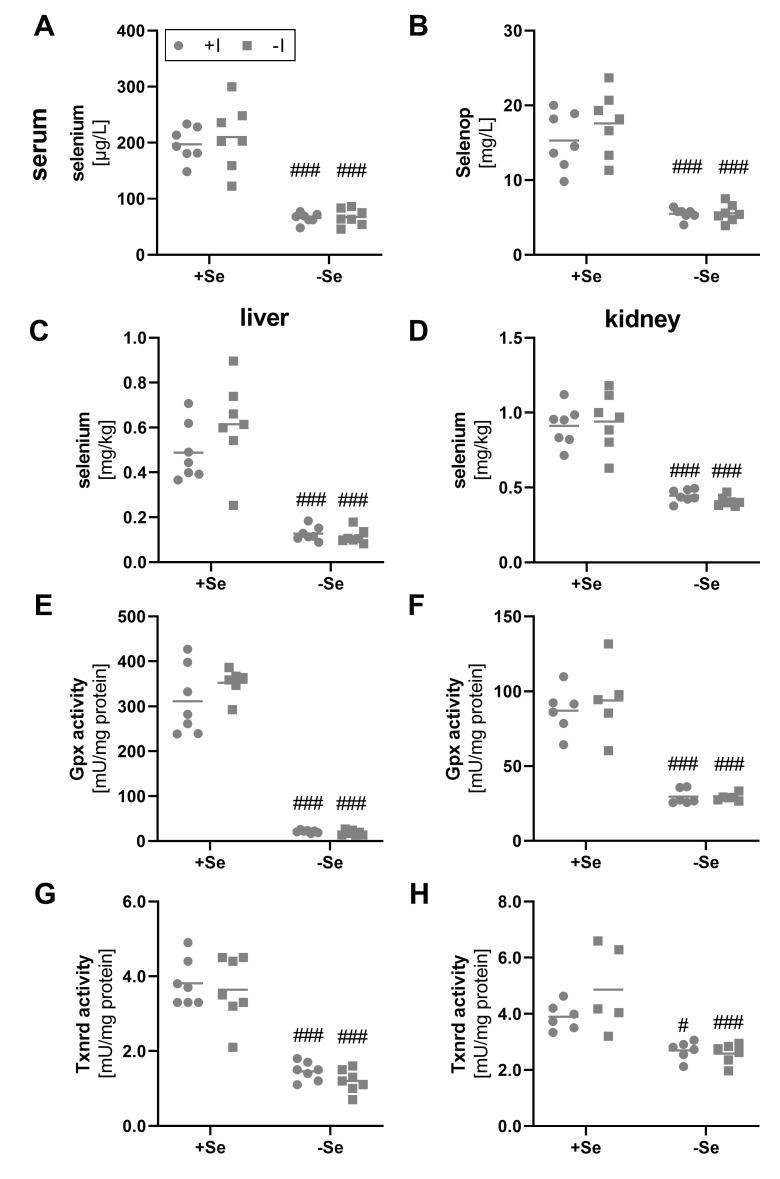
Biomarkers indicating selenium status in serum, liver, and kidney. To evaluate the selenium status, serum and tissue samples from male C57BL/6Jrj mice (n = 5–7) were used, which were supplied with either deficient or adequate amounts of selenium (Se) and iodine (I) for 8 weeks prior to sacrifice. Concentrations of selenium (**A**,**C**,**D**) and Selenop (**B**) were determined by ICP-MS/MS in serum (**A**,**B**), liver (**C**), and kidney (**D**). Further, selenium status was assessed by Gpx (**E**,**F**) and Txnrd activities (**G**,**H**), based on NADPH-consuming glutathione reductase-coupled assay and NADPH-dependent reduction of DTNB, respectively, in hepatic (**E**,**G**) and renal (**F**,**H**) tissue. Statistical analysis based on Two-way ANOVA and post hoc analysis using Bonferroni’s test with ^#^*p* < 0.05; ^###^*p* < 0.001 versus + selenium.

As not only the iodine but also the selenium supply was modulated within this feeding study, different biomarkers for the selenium status were analyzed as well. Total selenium content in serum was reduced by a factor of 3 in comparison to mice with an adequate selenium supply (Figure 2A). In parallel, the serum Selenop concentration was reduced in the selenium-deficient groups (Figure 2B). An influence of the varying iodine supply on serum status marker for selenium did not become evident.

Comparable results were observed by analyzing liver and kidney tissue. As expected, differences in the selenium supply resulted in a significant decrease of the total selenium content in the deficient groups (Figure 2C,D). This was also confirmed by significantly reduced hepatic and renal Gpx (Figure 2E,F) and Txnrd activities (Figure 2G,H). Differences in activity between the selenium-adequate and -deficient groups clearly exceeded the effects observed for mRNA expression, as hepatic and renal Gpx1 expression was only reduced to 0.5-fold in the selenium-deficient animals (Figure S2A,B), while almost no differences were found for hepatic and renal Txnrd1 expression between the experimental groups (Figure S2C, D). Again, none of the selenoproteins analyzed was affected by the iodine status of the mice.

### 3.3. Peripheral Effects of Thyroid Hormone in Response to a Low Iodine and Selenium Supply

**Figure 3:**
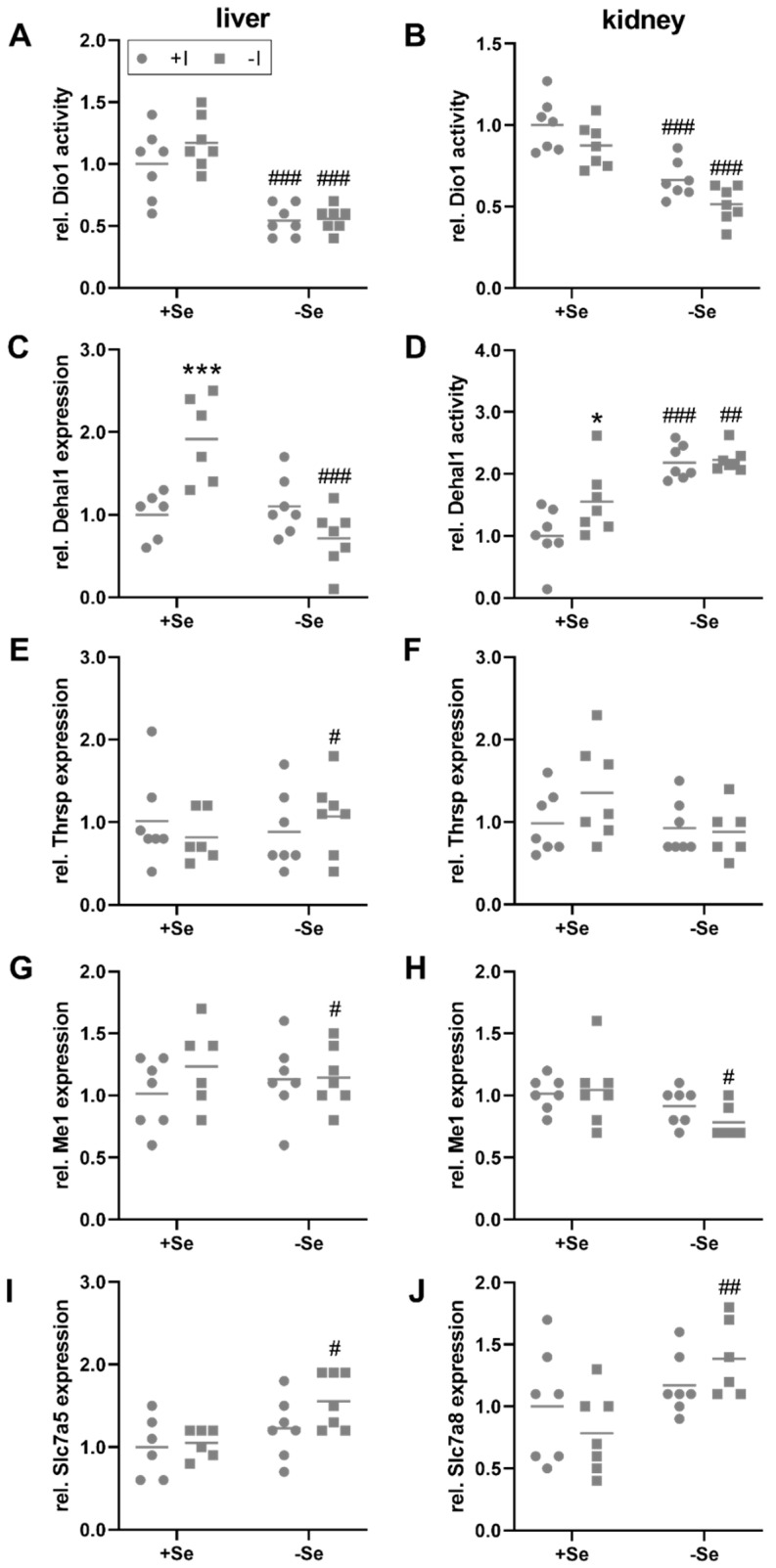
Local TH-induced effects in liver and kidney. To evaluate the impact of TH in liver (**A**,**C**,**E**,**G**,**I**) and kidney (**B**,**D**,**F**,**H**,**J**) of C57BL/6Jrj mice (n = 7, outliers excluded), supplied with either low or adequate amounts of selenium (Se) and iodine (I) for 8 weeks, enzyme activities (**A**,**B**,**D**) and gene expression (**C**,**E**–**J**) were assessed. Dio1 (**A**–**B**) and Dehal1 activity (**D**) were determined by Sandell-Kolthoff-based activity assays in liver (**A**) and kidney (**B**,**D**). Further, Dehal1 (**C**), Thrsp (**E**,**F**), Me1 (**G**–**H**), Slc7a5 (**I**), and Slc7a8 (**J**) expression was analyzed in liver (**C**,**E**,**G**,**I**) and kidney (**F**,**H**,**J**) by qRT-PCR. Expression levels were normalized to a composition factor based on the housekeeper genes Hprt, Rpl13a, 18S ribosomale RNA, beta-actin, TBP, and Gapdh. All results are expressed as fold change compared to +Se/+I-supplied animals. Statistical analysis based on Two-way ANOVA and post hoc analysis using Bonferroni’s test with * *p* < 0.05; *** *p* < 0.001 versus + iodine ^#^ *p* < 0.05; ^##^ *p* < 0.01; ^###^ *p* < 0.001 versus + selenium.

TH, bound to binding proteins, are distributed via the blood and enter peripheral organs, such as the liver and kidney, by TH transmembrane transporters, that are expressed in a cell type-specific manner. Subsequently, T4 is primarily converted into its more active form T3 by Dio1 expressed in liver, kidney, but also the thyroid itself. While thyroidal Dio1 activity was only responsive towards the iodine status (Figure 1C), hepatic and renal Dio1 activity was not modulated by the iodine supply but was significantly reduced in selenium-deficient animals (Figure 3A,B). However, no changes of Dio1 mRNA expression were observed in the liver and kidney in response to the intake of the two trace elements (Figure S2E,F). Hepatic mRNA expression levels of the iodine-recycling enzyme Dehal1 were increased by iodine deficiency but only in selenium-adequate mice (+Se/−I, Figure 3C). Under selenium deficiency, no iodine effect on Dehal1 expression was detectable. Besides this, the mRNA expression of target genes of the THR was analyzed. Hepatic Thrsp mRNA levels were slightly increased in selenium-deficient in comparison to selenium-adequate mice, but only under low iodine conditions (−Se/−I, Figure 3E). A comparable picture emerged for the expression of the THR target gene malic enzyme 1 (Me1), which was also affected by selenium under low iodine conditions only, and was slightly decreased in the animals with insufficient selenium supply (−Se/−I; Figure 3G).

The kidney is the main organ for iodine excretion. In comparison to the liver, partially different diet-induced effects were detected in the kidney. Renal Dehal1 activity could be measured. Irrespective of the iodine supply, a higher Dehal1 activity was found in animals that were insufficiently supplied with selenium (Figure 3D). Furthermore, under adequate selenium supply, a higher Dehal1 activity was detected in the iodine-deficient group. At the level of Dehal1 mRNA expression, no significant changes were found (Figure S2G). In contrast to the liver, the expression of Thrsp did not differ in any of the experimental groups (Figure 3F). Me1 expression was downregulated in -Se/-I-supplied animals (Figure 3H), as observed in the liver (Figure 3G).

In addition to the specific TH transporters monocarboxylate transporter 8 and 10, encoded by Slc16a2 and Slc16a10, some secondary transporters, such as the organic anion transporting polypeptide transporters (Oatp1a2, encoded by Slco1a2) and the large neutral amino acid transporters (Lat1 and Lat2, encoded by Slc7a5 and Slc7a8) are also involved in the uptake of TH [42]. Among them (Figure S3), hepatic Slc7a5 mRNA showed increased expression in -Se/-I-supplied group (Figure 3I). In the kidney, this effect was not observed for Slc7a5 but in this case for Slc7a8 (Figure 3J). At the expression level of THR, an increase was detected in liver for the expression of thyroid hormone receptor alpha (Thra) in selenium-deficient groups, which was complemented by a trend for an iodine effect (*p* = 0.078; Figure S3I). Accordingly, interactions between iodine and selenium do not appear to be limited to the level of selenoproteins but also include further proteins relevant for the TH axis.

### 3.4. Selenium and Iodine Deficiency Have Almost No Effect on the Status of Other Trace Elements

In order to evaluate possible effects of the varying selenium and iodine supply on the trace element homeostasis of iron, copper, and zinc, the total trace element contents and partly functional biomarkers, such as the iron transport protein transferrin were examined. In the serum, none of these parameters showed a variation depending on the trace element supply for selenium and iodine (Table 2). Also in the liver, the total iron, zinc, and copper levels were comparable between the groups. Only for copper a trend towards a reduced concentration in mice with adequate iodine supply was observed. In the kidney, however, the iron content was elevated with a low supply of selenium and the zinc content tended to be increased with a low iodine supply.

## 4. Discussion

Certain interactions of selenium and iodine with respect to the TH axis have been known for a long time [43], also the deficiency of other trace elements on TH synthesis is increasingly taken into account. However, to our knowledge, the perturbation of altered TH status by the nutritional supply of iodine and selenium and its effects on other trace elements is rarely subject to consideration.

Primary hypothyroidism, as triggered by iodine deficiency, is manifested by elevated TSH levels and reduced serum tT4 levels [44,45]. Both could be detected after eight weeks of dietary intervention in the C57BL/6Jrj animals in addition to significantly lowered serum iodine levels (Figure 1H, F). In contrast, tT3 levels remained unchanged (Figure 1G). This was unexpected but most probably tT3 will decrease only with progressive intervention as an indicator for advanced iodine deficiency. Interestingly, compared to all other groups including the +Se/+I-supplied group, +Se/−I supply led to a significant increase of tT3 serum levels. This is surprising but somehow in line with observations, that iodine deficiency can be accompanied by normal or even slightly elevated T3 as an immediate consequence of auto-regulatory mechanisms of the thyroid [46]. The maintenance of tT3 is in line with the lack of developmental impairments or altered organ weights observed in the animals, despite the immediate start of intervention after weaning.

Analysis of the thyroid glands clearly demonstrated that increase of serum TSH and subsequent receptor-mediated stimulation induced by iodine deficiency activated a variety of compensatory processes. Accordingly, Dio1, Dehal1, and peroxidase activity were increased in iodine-deficient animals (Figure 1B–D), representing activities involved in iodine recycling, TH metabolism, and TH biosynthesis, respectively. Thyroidal increase of Dio1 and Dehal1 are in line with the literature on effects of hypothyroidism in rodent models [47,48]. Especially for Dehal1 a recent study on molecular regulation in a rat thyroid cell line suggests an induction of the transcript by TSH and suppression by iodide and thyroglobulin [49]. Therefore, reduced iodine supply and resulting increase of TSH are the most likely mechanisms of thyroidal Dehal1 induction in our study, here demonstrated on the level of activity. Also, the tendency of increased total protein yield from thyroid lobes (data not shown) indirectly showed the enlargement of the gland by continuous TSH stimulation.

Due to iodine deficiency, the thyroid failed to produce and secret T4 in sufficient amounts (Figure 1F). T3, however, has a higher affinity for THR compared to T4 [3]. Consequently, the intracellular conversion of T4 to T3 by the selenoenzymes Dio is critical for the pre-receptor control of TH activity and downstream effects [50,51]. The liver and to some extent also the kidney play crucial roles in the peripheral metabolism of TH, but also for selenium distribution and excretion. Approximately 30 to 40% of the extrathyroidal T3 production originates from these two organs [52]. In this context, however, another characteristic emerges. Whereas the supply of selenium to the thyroid, like to the brain or testis, is maintained, the selenium content in the liver and kidney decreases faster in the case of persistently low selenium supply, according to the organ hierarchy of selenium [53]. The liver is thereby classified as more susceptible to selenium deficiency compared to the kidney [54]. Although the selenium content per mg of tissue is higher in the kidney, the difference in selenium content between adequately and deficient animals was much more pronounced in the liver (Figure 2C,D). This was also reflected by a stronger decrease in Gpx (16.6- vs. 3.0-fold) and Txnrd activities (2.8- vs. 1.6-fold) in liver compared to the kidney.

In general, the deiodinases are high in the hierarchy of selenoproteins [12,55]. Accordingly, thyroidal Dio1 activity was maintained independent of the selenium supply (Figure 1C). In the liver and kidney, Dio1 activity was reduced in selenium-deficient mice (Figure 3A,B). As Dio1 mRNA levels were not affected this is most probably a translational effect due to limited selenium availability and is in accordance with literature describing the organ-specific effects of severe selenium deficiency [56]. In comparison, Dehal1 activity was induced in selenium-deficient kidneys which might be an attempt to compensate for the reduced Dio activity, as Dio1 is also crucial in iodine retention to release it from TH metabolites prone to excretion via the bile. On the other hand, iodinated tyrosines (mono- and diiodothyrosine), side products of TH biosynthesis and thyroglobulin breakdown are secreted via the urine. Hence, increased renal Dehal1 activity is a meaningful step to retain and rescue the bound iodine in situations of deficiency. For full interpretation, it has to be kept in mind, that function (but also determination) of Dehal1 activity relies on an unknown endogenous NADPH-dependent reductase activity [34]. Regulation of the latter one could also affect activity, independent from any transcriptional changes.

Renal Dio1 activity was also moderately affected by iodine status, which was independent of the selenium supply (Figure 3B). Iodine deficiency led to a mild decrease in activity, which is consistent with data from hypothyroid animals [57]. Again, Dehal1 activity was counter-regulated (increased by iodine), but only in +Se/−I-supplied animals (Figure 3D). Comparable counter-regulation of Dio1 and Dehal1 has been reported from hyperthyroid animals [34]. In liver, Dehal1 activity was below the detection limit, but mRNA expression was also increased in +Se/−I-supplied mice (Figure 3C). This pattern is in line with the increased circulating tT3 concentrations in animals of the +Se/−I-supplied group (Figure 1G). Others have described a decrease of hepatic Dehal1 transcripts in situations of chronic iodine deficiency in rats [48]. While the selenium status of these animals was not reported, this discrepancy also demonstrates that there is a need for further studies to understand physiological Dehal1 regulation under different conditions. Thus, although Dehal1 is not a selenoprotein, its expression and activity is modulated by the selenium and iodine status.

TH exert their effect via interaction with nuclear THR, which regulate the expression of numerous target genes. It is estimated that 3% to 13% of hepatic genes are regulated by T3 [58,59]. These include Dio1, Thrsp [60,61], important in the regulation of lipid metabolism, but also Me1 [62,63], generating NADPH for fatty acid biosynthesis. As serum tT3 levels were still maintained (Figure 1G), it was not surprising that no iodine-dependent regulation of the expression of these genes could be detected. Nevertheless, the analysis of hepatic Thrsp (Figure 3E) and Me1 (Figure 3G, H) indicated that a combined selenium and iodine deficit (−Se/−I) was necessary to induce an altered expression. In line with this notion, the observed upregulation of TH transporters (Figure S3I) in the -Se/-I-supplied group appears to be a combinatory effect induced by low T4 levels in response to low iodine and reduced local Dio1 activity due to selenium deficiency. Thus, at least in mice, interactions of iodine and selenium with regard to the TH axis are more pronounced in TH target organs than in the thyroid itself.

Other trace elements are also associated with TH biosynthesis, metabolism, and action; among them are iron, zinc, and copper (e.g., [20,30,64]). A study based on data of the National Health and Nutrition Examination Survey (NHANES) showed a positive correlation of serum copper levels with free and total T4 in women and tT4 and tT3 in men, and a negative correlation of fT4 and tT4 with zinc in men [65]. The association of copper and selenium with fT4 has also been suggested elsewhere [66]. Others, however, observed increased blood concentrations of copper and zinc in hypothyroid patients [67], while a recent meta-analysis concluded that hypothyroidism is associated with lower serum selenium and zinc levels [68]. But also no association between levels of copper, selenium, or zinc with TH have been observed [69]. Although the molecular mechanisms for the involvement of the trace elements in TH homeostasis are widely understood, obviously human data from different sources (with the exception of selenium) are rather contradictory. This may be due to the fact, that in human studies only in rare cases drastic differences in TH levels are observed and that iodine and selenium supply varies considerably worldwide. A study in mice on interactions of copper and TH stated that TH increase serum copper concentrations through concerted regulation of various transporters and binding proteins in the liver and kidney [30]. Herein, the effect of T3 on corresponding correlating variables was shown. These have also been partially demonstrated for T4. However, although serum tT4 levels were significantly modulated in this experiment (Figure 1F), tT3 levels were barely altered (Figure 1G). This may be an explanation that despite significant alterations in iodine, selenium, and TH metabolism and balance, no drastic changes were detected at the level of other trace elements, at least in serum. However, as the intervention-induced effect on iodine homeostasis was much stronger compared to a large number of human studies, an alternative conclusion could be that the influences on other trace elements are not as severe as other studies suggest.

Nevertheless, a recent human study observed inverse correlations between free T4 and the copper/selenium ratio [69]. Looking at correlations between TSH, tT3, and tT4 within our study, in addition to the expected associations between these three serum parameters and markers of thyroidal function and activation (Dio1, Dehal1, and peroxidase activity as well as iodine content of the thyroid tissue), there are also positive correlations for tT3 with copper serum levels (r_s_ = −0.414) as well as negative correlations for tT3 with copper contents in the kidney (r_s_ = −0.408). These correlations were not overly strong and only barely reached significance level. The extent to which attention should be paid to them would need to be investigated in studies with larger numbers of animals. Furthermore, such studies should aim towards additional lowering of T3 levels (potentially by longer intervention) to better understand the possible interactions of TH and essential trace elements, such as selenium, copper, iron, and zinc. 

## 5. Conclusions

Iodine and selenium play an essential role in the production of TH. In this regard, this study conclusively shows that thyroid and TH homeostasis are quite robust to selenium deficiency compared to TH target organs such as liver and kidney. However, from a practical point of view, it is also clear that in animal studies focusing on selenium- or iodine-dependent effects, the nutritional status should be reported for both elements for better interpretation. Also, further consideration of possible interactions between TH and copper in particular is needed. But also other essential trace elements such as iron and zinc and their possible effects on TH should be further studied as trace element deficiencies rarely occur alone, making their interactions very relevant for human health.

## Figures and Tables

**Table 1:** TE supply of mice. Feeding recommendations [31] and content of the trace elements selenium (Se), iodine (I), iron (Fe), copper (Cu), manganese (Mn), and zinc (Zn) in food and water within the animal experiment. Feed contents were determined by ICP-MS/MS. * supplied concentration below recommendation, but substantially above a concentration that triggers a deficiency.

Elements	Feeding Recommen-dations [mg/kg]	TE Content of Feed [mg/kg]	TE in Drinking Water [mg/kg]				Total Supply [mg/kg]			
			+Se/+I	+Se/−I	−Se/+I	−Se/−I	+Se/+I	+Se/−I	−Se/+I	−Se/−I
Se	0.15	0.02	0.13	0.13	-	-	0.15	0.15	0.02	0.02
I	0.15	0.03	0.15	-	0.15		0.18	0.03	0.18	0.03
Cu	6.00	3.65	-	-	-	-	3.65 *	3.65 *	3.65 *	3.65 *
Fe	35.0	17.2	17.8	17.8	17.8	17.8	35.0	35.0	35.0	35.0
Mn	10.0	9.65	-	-	-	-	9.65	9.65	9.65	9.65
Zn	30.0	46.2	-	-	-	-	46.2	46.2	46.2	46.2

**Table 2:** Impact of low or adequate selenium and iodine supply on iron, zinc, and copper homeostasis. Concentrations of trace elements or functional markers for iron (Fe), zinc (Zn), and copper (Cu) in serum, liver, and kidney detected by ICP-MS and ELISA in male C57BL/6Jrj mice (*n* = 7). Data are presented as mean ± standard deviation. Statistical testing based on Two-Way ANOVA and post hoc analysis using Bonferroni’s test with * *p* < 0.05. n.s. not significant.

Tissue	Element [Unit]	+Se/+I	+Se/−I	−Se/+I	−Se/−I	Se Effect	I Effect
serum	Fe [µg/L]	1916 ± 572	2277 ± 1093	2049 ± 341	2260 ± 409	n.s.	n.s.
	transferrin [mg/mL]	2.1 ± 0.2	2.4 ± 0.6	2.2 ± 0.2	2.3 ± 0.3	n.s.	n.s.
	Zn [µg/L]	711 ± 126	680 ± 158	689 ± 138	773 ± 85.1	n.s.	n.s.
	Cu [µg/L]	428 ± 92.7	351 ± 58.7	353 ± 95.1	433 ± 127	n.s.	n.s.
liver	Fe [mg/kg]	72.3 ± 10.5	67.6 ± 17.5	70.8 ± 8.5	66.7 ± 9.3	n.s.	n.s.
	Zn [mg/kg]	27.1 ± 4.1	27.8 ± 2.8	27.1 ± 3.1	25.8 ± 1.7	n.s.	n.s.
	Cu [mg/kg]	4.6 ± 0.3	4.8 ± 0.3	4.6 ± 0.4	4.9 ± 0.3	n.s.	0.088
kidney	Fe [mg/kg]	84.2 ± 4.7	77.0 ± 8.5	87.7 ± 8.1	89.5 ± 12.9	*	n.s.
	Zn [mg/kg]	21.5 ± 1.0	22.3 ± 0.7	22.2 ± 0.5	22.9 ± 1.5	n.s.	0.053
	Cu [mg/kg]	5.1 ± 0.3	4.9 ± 0.3	4.9 ± 0.3	5.1 ± 0.4	n.s.	n.s.

## Supplementary Materials

The following are available online at https://www.mdpi.com/article/10.3390/nu13113773/s1, Figure S1: Physiological characteristics after dietary intervention, Figure S2: Impact of low or sufficient selenium and iodine supply on selenium-dependent marker. Figure S3: Influence of selenium and iodine supply on the expression of transporters and receptors for TH. Table S1: Oligonucleotide sequences.
