# Supplementary material for: The Nutritional Supply of Iodine and Selenium Affects Thyroid Hormone Axis Related Endpoints in Mice
Source: Nutrients. 2021 Oct 25;13(11):3773. doi: 10.3390/nu13113773 (PMC8625755; doi:10.3390/nu13113773)
Supplement: Supplementary file 1 [file nutrients-13-03773-s001.zip › nutrients-1418262-supplementary.pdf]

The nutritional supply of iodine and selenium affects thyroid hormone axis related endpoints in mice

Kristina Lossow, Kostja Renko, Maria Schwarz, Lutz Schomburg, Tanja Schwerdtle, Anna Patricia Kipp

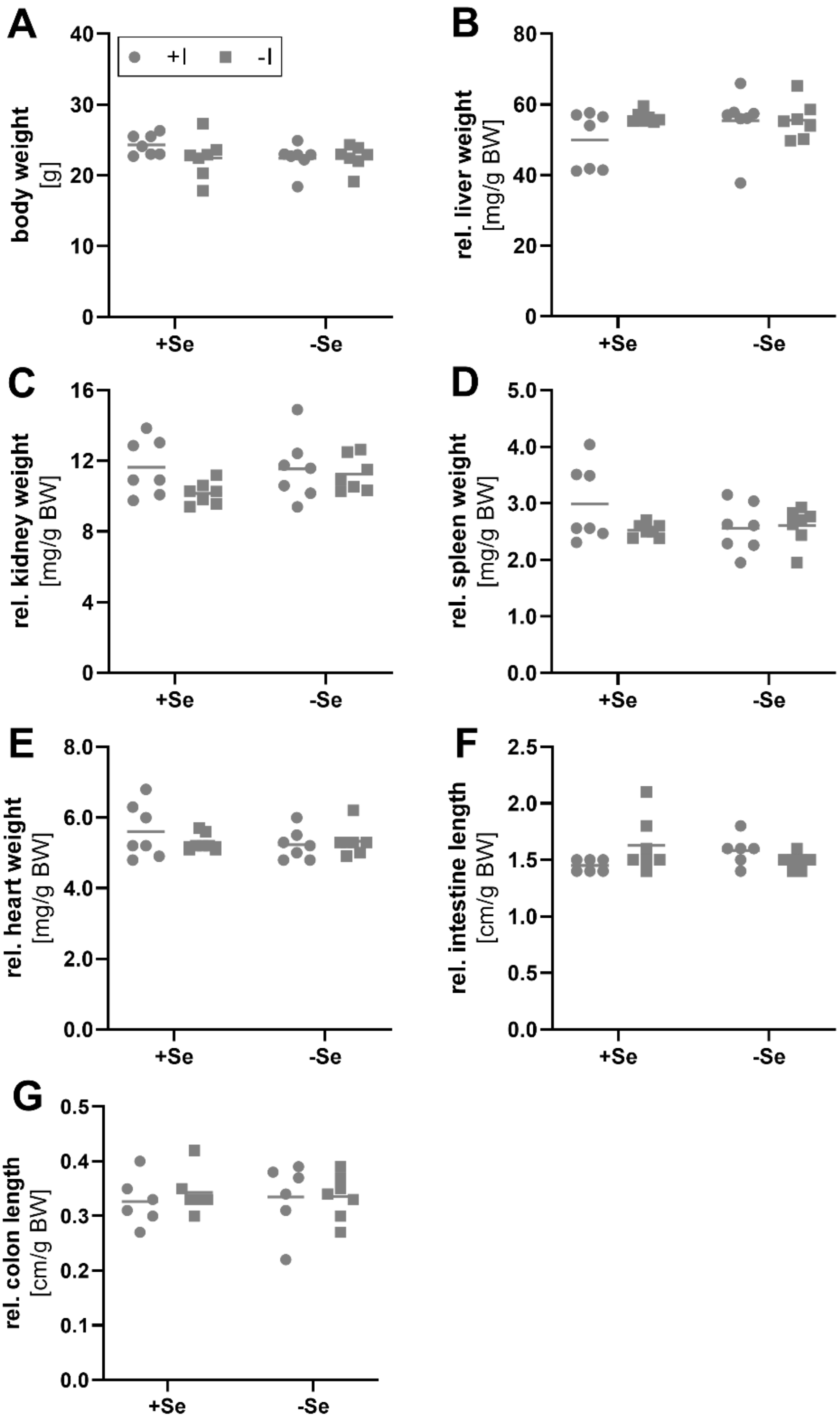

Figure S1. Physiological characteristics after dietary intervention. Body weight and relative organ weight of liver (B), kidney (C), spleen (D), and heart (E) as well as the relative length of the small intestine (F) and colon (G) based on data from 7 C57BL/6Jrj mice after 8 weeks of dietary intervention with varying selenium and iodine supply. Statistical analysis based on two-way ANOVA, but no differences were found.

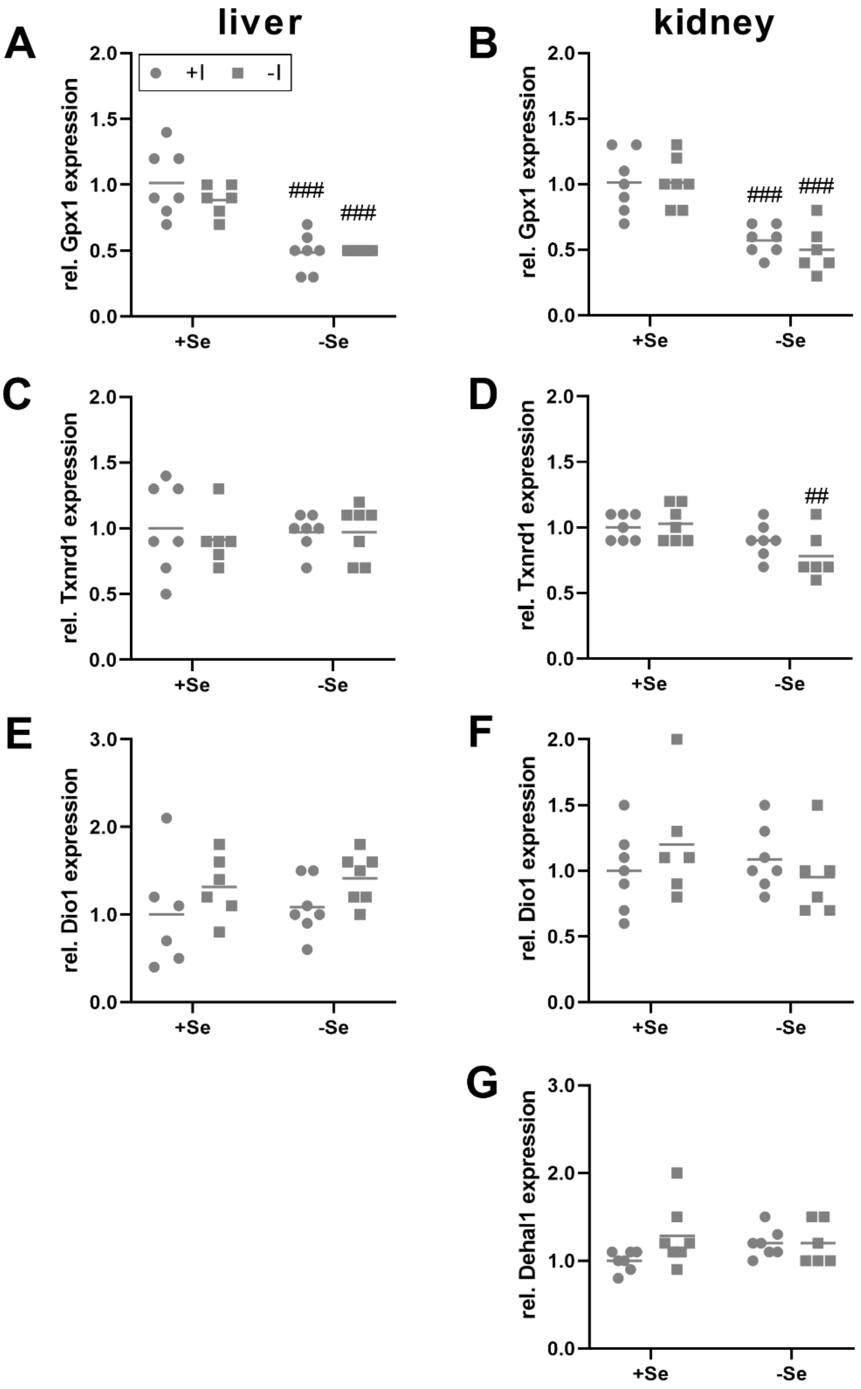

Figure S2. Impact of low or sufficient selenium and iodine supply on selenium-dependent marker. Detection of various selenium-dependent quantities in liver (A, C, E,) and kidney (B, D, F, G) by analysis of gene expression of 7 C57BL/6Jrj animals after varying intake of selenium and iodine. Messenger RNA expression analysis of the selenoproteins glutathione peroxidase (Gpx1; A, B), thioredoxin reductase 1 (Txnrd1; C, D), and diodinase 1 (Dio1; E, F) was considered for qRT-PCR. Complemented with expression data for iodine-releasing iodotyrosine deiodinase (Dehal1) in kidney (G).

Expression levels were normalized to a composition factor based on the housekeeper genes Hprt, Rpl13a, 18S ribosomale RNA, beta-actin, TBP, and Gapdh. Expression variances are expressed as fold change compared to + Se/+ I supplied animals. Statistical testing based on Two-Way ANOVA and post hoc analysis using Bonferroni´s test with $^{\#}p < 0.05$; $^{\#\#}p < 0.01$; $^{\#\#\#}p < 0.001$ versus + selenium.

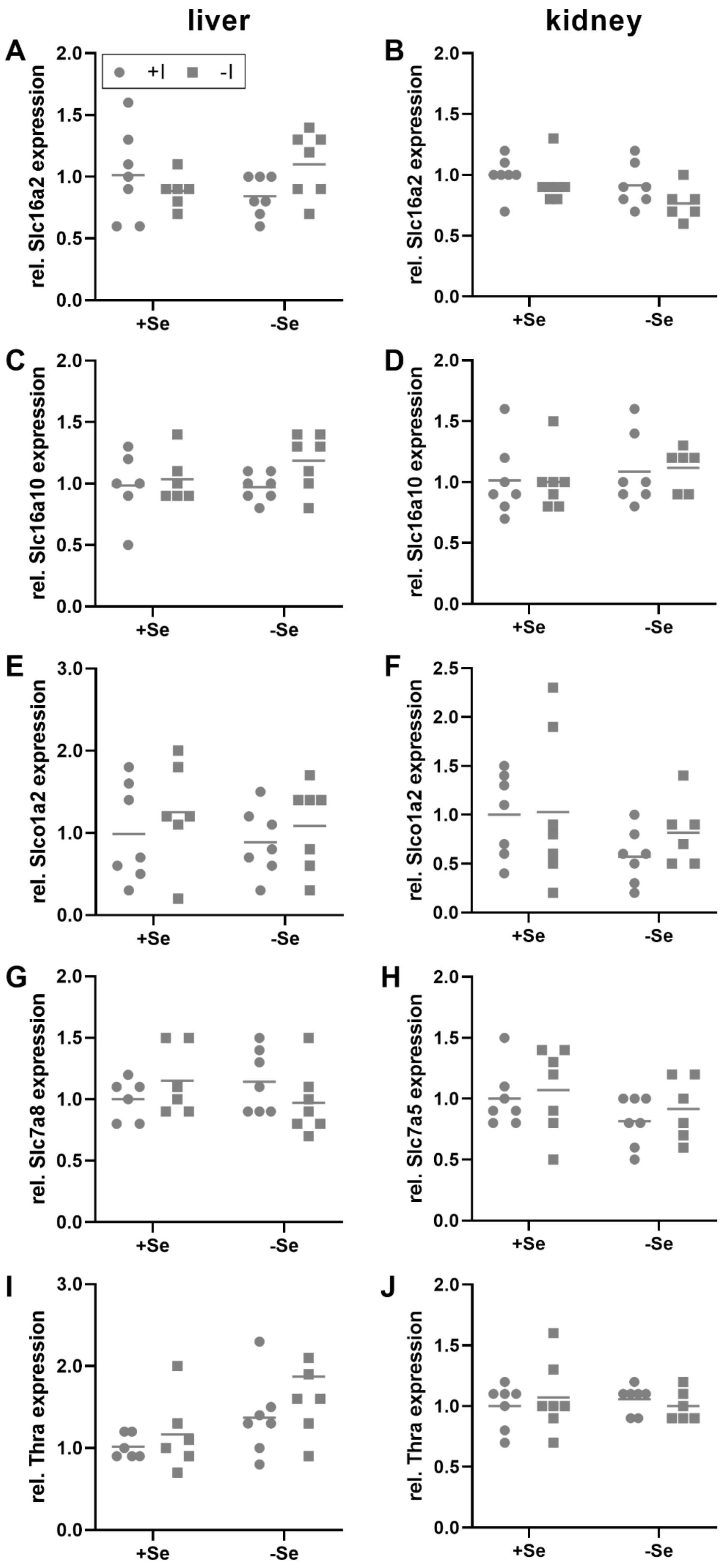

liver
kidney
A
B
C
D
E
F
G
H
I
J
+I
-I
rel. Slc16a2 expression
rel. Slc16a10 expression
rel. Slco1a2 expression
rel. Slc7a8 expression
rel. Slc7a5 expression
rel. Thra expression
+Se
-Se

Figure S3. Influence of selenium and iodine supply on the expression of transporters and receptors for thyroid hormones. In the livers (A, C, E, G, I) and kidneys (B, D, F, H, J,) of 7 C57BL/6Jrj animals, gene expression of various target genes was determined by qRT-PCR analysis after varying selenium and iodine supply over a period of 8 weeks. The target genes included the transporters of thyroid hormones like the monocarboxylate transporter 8 and 10, encoded by Slc16a2 (A, B) and Slc16a10 (C, D), respectively, organic anion transporting polypeptides 1a2, encoded by Slco1a2 (E, F) or the large neutral amino acid transporters LAT1 and LAT2, encoded by Slc7a5 (H) and Slc7a8 (G), respectively. Additionally, expression of thyroid hormone receptors a (Thra; I, J) was evaluated. Expression levels were normalized to a composition factor based on the housekeeper genes Hprt, Rpl13a, 18S ribosomale RNA, beta-actin, TBP, and Gapdh. Expression variances are expressed as fold change compared to + Se/+ I supplied animals. Statistical testing based on Two-Way ANOVA and post hoc analysis using Bonferroni´s test with $^{\#}p < 0.05$; $^{\#\#}p < 0.01$ versus + selenium.

Table S1. Oligonucleotide sequences (5′→3′).

gene	RefSeq-ID	sequence	Tm [°C]
18S ribosomale RNA	NR_003278	GAATTCCCAGTAAGTGCGGG	60
		GGGCAGGGACTTAATCAACG	
beta-actin	NM_007393.3	CACTGCCGCATCCTCTTCCT	60
		GATTCCATACCCAAGAAGGAAGGC	
Dio1, diodinase 1	NM_007860.3	GGGATTTCATTCAAGGCAGCAGG	60
		TGTGGAGGCAAAGTCATCTACGA	
Dio2, diodinase 2	NM_010050.3	GTGTCTGGAACAGCTTCCTCCT	60
		CCATCAGCGGTCTTCTCCGA	
Gapdh, glyceraldehyde 3-phosphate dehydrogenase	NM_199472	GGGTGTGAACCACGAGAAAT	60
		GTCTTCTGGGTGGCAGTGAT	
Gpx1, glutathione peroxidase 1	NM_008160.5	GAAGAGATTCTGAATTCCCTCAA	60
		GAACTTCTCAAAGTTCCAGGCA	
Hprt1, hypoxanthine guanine phosphoribosyl transferase 1	NM_013556.2	GCAGTCCCAGCGTCGTG	60
		GGCCTCCCATCTCCTTCAT	
IYD, iodotyrosine deiodinase	NM_027391.4	GACCTGAAGAAACTGAGAACCA	50
		TTTCCATTCGCAGCAAAACC	
Me1, malic enzyme 1	NM_001198933.1	CCACAACAGTGTCTACCCAT	50
		TCATCCAGGAAGGCGTCATA	
Rpl13a, ribosomal protein L13a	NM_009438.5	GTTCGGCTGAAGCCTACCAG	60
		TTCCGTAACCTCAAGATCTGCT	
Slc7a5, solute carrier family 7, member 5	NM_011404.3	TAAAGGCTGCGACCCGTGTG	60
		ACGCATCACCTTGTCCCATGTC	
Slc7a8, solute carrier family 7, member 8	NM_016972.2	CCTGCTCTTCACATGCCTCTC	60
		ATCTGTCCTGCAACCGTTACCC	
Slc16a2, monocarboxylic acid transporter member 2	NM_009197.2	CTTCACCAGCTCCCTAAGCCT	60
		AGTAGTGGCCCAGGATGACGA	
Slc16a10, monocarboxylic acid transporter member 10	NM_001114332.1	GCCTTTGAACTAGTTGGTCCTC	60
		ATGAAGTAACCCTGCAATAGGAGG	
Slco1a2, solute carrier organic anion transporter family member 1a2	NM_013797.5	TAGCTTGCCTCCAGTATGCCTT	60
		GACAGGCCAAATGCTATGTATGC	
TBP, TATA box binding protein	NM_013684.3	ACCGTGAATCTTGGCTGTAAAC	60
		GCAGCAAATCGCTTGGGATTA	
Thra thyroid hormone receptor alpha	NM_001313983.1	GGAGAACAGTGCCAGGTCACCA	60
		TGATAACCGGTGGCCTTGTCCC	
Thrsp, thyroid hormone responsive spot 14	NM_009381.3	AGGTGACGCGGAAATACCAG	60
		TCTCTCGTGTAAAGCGATCTTCAG	
TSH beta, thyroid-stimulating hormone	NM_009432.2	GGGTATTGTATGACACGGGATA	60
		ATTTCCACCGTTCTGTAGATGA	

**gene**	**RefSeq-ID**	**sequence**	**Tm [°C]**
Txnrd1, thioredoxin reductase 1	NM_001042523.1	AGCAGCTAAGGAGGCAGCCA	60
		TTTCCAGCCATAGTTGCGCGAG	
